# An interleukin-1 polymorphism additionally intensified by atopy as prognostic factor for aseptic non-mechanical complications in metal knee and hip arthroplasty

**DOI:** 10.3389/fimmu.2022.1050315

**Published:** 2022-11-28

**Authors:** B. Summer, D. Lill, K. Remmel, A. Schraml, C. Schopf, I. J. Banke, H. Kuechenhoff, T. Maierhofer, S. Endres, P. Thomas

**Affiliations:** ^1^ Department of Dermatology and Allergology, University Hospital, Munich, Germany; ^2^ Department of Orthopedic Surgery, Clinic Rummelsberg and Nürnberg, Rummelsberg, Germany; ^3^ Department of Orthopaedics, Physical Medicine and Rehabiliation University Hospital, Munich, Germany; ^4^ Clinic of Orthopedics and Sports Orthopedics, Klinikum rechts der Isar, Technical University of Munich (TUM), Munich, Germany; ^5^ Statistical Consulting Unit StaBLab, Department of Statistics, LMU, Munich, Germany; ^6^ Department of Statistics, University of California, Los Angeles (UCLA), Los Angeles, CA, United States; ^7^ Division of Clinical Pharmacology, University Hospital, Munich, Germany

**Keywords:** genetics, polymorphism, interleukin-1, arthroplasty, complications, adverse reaction, allergy, atopy

## Abstract

**Background:**

In contrast to infection or mechanical issues joint replacement failure following inflammatory adverse reactions is poorly understood.

**Objective:**

To assess the association of IL-1β polymorphisms and history of allergy with aseptic non-mechanical complications following arthroplasty.

**Methods:**

In 102 patients with aseptic non-mechanically caused symptomatic knee or hip arthroplasty (SA) and 93 patients with asymptomatic arthroplasty (AA) questionnaire-based history, patch test with at least standard series, lymphocyte transformation test (LTT) with nickel, cobalt and chromium and interleukin-1 polymorphism analysis were done. Three polymorphisms of the IL1B gene [IL-1b -3954 (rs1143634), IL-1b -511 (rs16944) and IL-1b -31 (rs1143627)] and one polymorphism of the IL1RN gene [IL1RN intron 2, variable number of tandem repeats, VNTR (rs2234663)] were assessed by PCR and gel electrophoresis.

**Results:**

We found no significant difference in smoking history and atopy but 25% versus 10% of self-reported metal allergy in SA versus AA; the patch test (respective, LTT) for metal sensitivity was more often positive in SA patients. The allele 498 bp of the IL1RN polymorphism occurred significantly more often in the SA group (37% versus 11%; p < 0.0001). Upon additional presence of atopy, the difference was even greater (60% vs 10%) (p < 0.000001). There was no association of IL-1 polymorphisms with metal allergy.

**Conclusion:**

The IL1RN VNTR allele 498 bp was strongly associated with SA. In patients with a history of atopy, presence of the IL1RN VNTR allele 498 bp led to a four-fold higher SA prevalence compared to patients without this allele.

## Introduction

A continuously increasing number of metallic devices is implanted worldwide. In 2011 the numbers of performed total hip replacement (THR) and knee replacement (TKR) were 465.034 THR and 702.415 TKR in the United States respectively 232.320 THR and 168.486 TKR in Germany (1) – and about 9% of these were complication-related revision surgery. For 2030 in the United States a two-time growth for THR and a five-time growth for TKR is anticipated with corresponding increase of revision surgeries (Lit Schwartz 2020) (2). In Europe a similar growth is anticipated with greater increase of THRs than TKRs. The considerable revision rate not only concerns a large patient group but in general, about 5% of patients with THR and up to 20% of patients with TKR are unsatisfied with its outcome (3). A meta-analysis of the different joint replacement registers has shown that apart from infections and mechanical reasons the so called (aseptic) adverse reactions to arthroplasty materials account for a majority of revision surgeries (4). The umbrella term ,,adverse reaction “reflects a spectrum of clinical symptoms resulting from altered or exaggerated inflammatory response to the implant as ,,foreign body “. Local and widespread dermatitis or periimplant lymphocytic infiltrates in connection with metal allergy can indicate a specific hyperreactivity (5). However, as Munch et al. demonstrated by cross-matching the Danish contact dermatitis and arthroplasty registries, there is no clear link between failed arthroplasty and metal allergy being proven in parallel. At least, in this study metal allergy was more frequent in patients with multiple revisions i.e. repeated implant failure (6). To better understand the role of metal allergy as elicitor of complications further studies are needed (7). The often-unspecific symptoms of adverse reactions to metal arthroplasty include pain, swelling, recurrent effusions, reduced range of motion, instability due to loosening. The most studied scenario is reactivity to wear particles that generates periprosthetic osteolysis leading to aseptic loosening. Amount and type of particles but also individual susceptibility may modulate such response. It was shown, that peripheral blood phagocytic cells from different human donors vary in their cellular osteolytic response to wear particles (8) suggesting different osteolysis susceptibility. Candidate gene analysis has shown nucleotide polymorphisms in several proinflammatory cytokines or regulatory elements such as RANK or TIRAP (9, 10). However, other studies found no association between aseptic loosening and for example BCL2 or CALCA genes (11). In 2020 Koks et al. reported about their genome wide association study, in which SNP´s in several genes were found to be associated with aseptic loosening, i.e. with shorter time span to revision surgery (12). In a broader sense, interleukin-1 gene cluster polymorphisms are found in many different clinical conditions with increased inflammatory manifestation (13, 14). Among the most studied are the IL-1B-gene and the IL-1RA-gene. With regard to knee osteoarthritis few studies described associated single nucleotide polymorphisms within the IL-1 gene cluster, but their significance is not fully clear. As summarized in a systematic review by Budhiparama et al. (15) IL-1RN alleles – for example IL-1RN*2 allele - might be associated with severity of knee osteoarthritis. However, the role of genetic susceptibility in metal arthroplasty adverse reactions is unclear. Since several years we have been running an ambulatory clinic for patients with suspected intolerance/allergic reactions to metal implants. Most of them are presenting with otherwise unexplained symptoms to TKR. Our allergy diagnostics includes patch testing to allergen series containing metal and bone cement components, supplemented by metal-LTT. In order to investigate the potential role of genetic susceptibility in addition to allergy in these patients, we thus aimed to (i) perform the examinations by including symptom free arthroplasty patients, (ii) focus on the IL-1B-gene with the polymorphisms IL-1B-3954 (rs1143634), IL-1B-31(rs1143627), IL1B-511(rs16944) and one mini satellite polymorphism (IL-1RN Intron 2 VNTR, rs2234663) and (iii) further evaluate the results by including the atopy - background and test results of the patients.

## Methods

### Study design and participants

**Figure 1:**
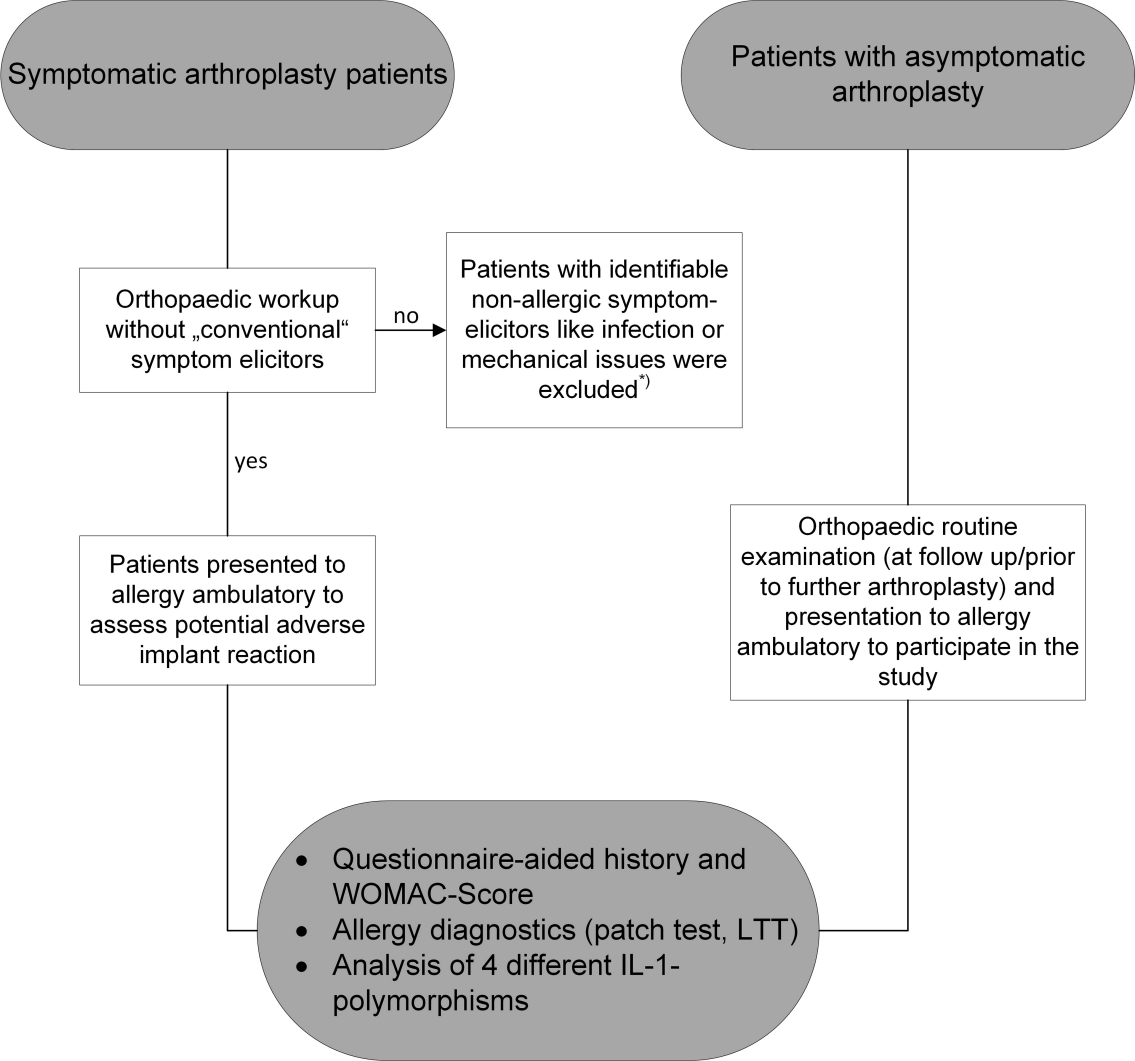
Schematic of the study design. The initial work-up for symptomatic arthroplasty was done by the treating orthopaedic surgeon. Once “conventional” problem elicitors like infection or mechanical issues were ruled out, patients were presented for allergy diagnostics and asked for consent to additional subsequent polymorphism analysis.*= i.e. therapy was performed according to the identified complication elicitors by the treating orthopaedic surgeons.

195 patients with TKR or THR took part in the study after giving their informed consent. This study was approved by the local ethics committee. The study was registered with ClinicalTrials.gov, number NCT02262065. The methods included questionnaire-aided procedures, patch testing, LTT and polymorphism analysis. **Figure 1** gives a schematic of the study design.

In all patients at least some component was Cobalt-Chromium-Molybdenum-(CoCrMo-)-based. Patients with asymptomatic arthroplasty (AA) were individuals with well-functioning arthroplasty being looked after in the orthopaedic clinics of Rummelsberg, LMU and TUM. They were either seen during routine follow up or because another arthroplasty (contralateral or different joint region) was planned. Patients with symptomatic arthroplasty (SA) had been sent by various referring orthopaedic surgeons to the allergy department after a primary clinical and radiographic orthopaedic examination to exclude elicitors of complaints like periimplant infection or mechanical problems for example like malpositioning. The presentation to our ambulatory was for evaluation of implant intolerance, namely implant allergy. A consecutive series of such patients was asked to participate in the study. All four sites reported patient history by use of the same questionnaire-aided procedure and patch test reactivity was done with the same manufacturer’s materials and test protocols. Pseudonymized blood samples were processed in one single laboratory to avoid the risk of evaluation error due to different LTT and PCR assays.

Theoretically, some selection bias may have happened in the group of symptomatic patients, since firstly patients with self-reported history of suspected metal allergy may have preferably been referred and secondly the background numbers of dissatisfaction or symptomatic arthroplasty is generally higher in TKR than in THR. In total, 102 patients (72f, 30m, 41-81yrs; 98 with TKR and 4 with THR) had SA. The control group consisted of 93 patients with AA (70f, 23m, 18-96yrs; 41 with TKR and 52 with THR).

### Questionnaire

First, a questionnaire-aided history was taken including information about smoking, medication, implant type, history of metal allergy and history of atopic diseases such as allergic rhinitis, atopic eczema or asthma.

Second, the orthopaedic WOMAC-questionnaire (the Western Ontario and McMaster Universities Arthritis Index, WOMAC) was used to quantify the respective patient´s self-administered view of the artificial joint performance covering items for pain, stiffness and functional limitation (16).

### Allergens and patch testing

All of the patients were patch tested. For this Almirall-Hermal patch test preparations and Finn-chambers on Scanpor (Almirall Hermal, Reinbek, Germany) were used. The substances were applied on the upper back on D0. The standard series with 29 allergens (including Nickel (Ni), Co and Cr preparations), a routine supplemental series and a bone cement series (if the respective patients had a cemented arthroplasty) was tested according to the German Contact Dermatitis Research Group (DKG) guidelines. Readings were done by physicians of the allergy unit on day 2 and 3. The reactions reported as positive in this paper were reactions classified as +, ++ and +++.

### Lymphocyte transformation test

LTT was performed as published by Stander and Summer (17). Shortly, peripheral blood mononuclear cells (PBMC) were isolated by density centrifugation and stimulated in quadruplicate over 6 days with different concentrations of NiSO4, CoCl2 or CrCl3. Control stimuli were the T-cell mitogen phytohemagglutinine (PHA, Biochrom, Berlin, Germany) 2.4 μg/ml, tetanus toxoid as control recall antigen (TT, Chiron Behring, Berlin, Germany) 5 μg/ml. After 5 days the PBMC were pulsed with 3H thymidine and proliferation was assessed after overnight incubation by measurement of the incorporated radioactivity. The results are given as stimulation index (SI) which means the ratio of incorporated radioactivity in stimulated versus non-stimulated control cultures. An SI≥3 was considered as a positive reaction.

### Polymorphism analysis

DNA was isolated from the patients peripheral blood with the ,,peqGOLD Blood DNA Mini Kit” (Peqlab, Erlangen, Germany) according to the protocol. The obtained DNA was diluted with distilled H2O to a final concentration of 40 ng/µl. The three polymorphisms IL-1B-3954 (rs1143634), IL-1B-31(rs1143627), IL1-B-511(rs16944) and the mini satellite polymorphism IL-1RN Intron 2 VNTR (rs2234663) were analysed by PCR, digest with restriction enzymes TaqI, Alu1 or Ava1 followed by gel electrophoresis.The DNA sequences of the polymorphisms are given in **Table 1**.

**Table 1:** The analyzed polymorphisms with the respective DNA sequences.

Polymorphism	DNA sequence
**IL-1B-3954** **(rs1143634)**	CTCCACATTTCAGAACCTATCTTCTT[C/T]GACACATGGGATAACGAGGCTTATG
**IL-1B-511** **(rs16944)**	CTACCTTGGGTGCTGTTCTCTGCCTC[A/G]GGAGCTCTCTGTCAATTGCAGGAGC
**IL-1B-31** **(rs1143627)**	AGCCTCCTACTTCTGCTTTTGAAAGC[C/T]ATAAAAACAGCGAGGGAGAACTGG
**IL-1RN** **Intron 2 VNTR (rs2234663)**	ACTCCTATTGACCTGGAGCACAGGT[(ATCCTGGGGAAAGTGAGGGAAATATGGACATCACATGGAACAACATCCAGGAGACTCAGGCCTCTAGGAGTAACTGGGTAGTGTGC)2/3/4/5/6]TTGGTTTA

The polymorphisms with their respective restriction enzyme and the resulting fragments are given in **Table 2**.

**Table 2:** The analyzed polymorphisms with the respective restriction enzymes and the resulting DNA fragment lengths.

Polymorphism	Restriction enzyme	Fragment length (bp)
**IL-1B-3954** **(rs1143634)**	Taq I	T: 250 bp C: 136 bp + 114 bp
**IL-1B-31** **(rs1143627)**	Alu 1	C: 281 bp T: 184 + 97 bp
**IL-1B-511** **(rs16944)**	Ava 1	T: 304 bp C: 189 bp + 115 bp
**IL-1RN** **Intron 2 VNTR (rs2234663)**	Ø	Allele 1: 412 bp (4 repeats) Allele 2: 240 bp (2 repeats) Allele 3: 498 bp (5 repeats) Allele 4: 326 bp (3 repeats) Allele 5: 584 bp (6 repeats)

### Association analysis

The prespecified primary endpoint was the occurrence of the here assessed three polymorphisms of the IL1B gene and one of the IL1RN with regard to asymptomatic or symptomatic, complicated arthroplasty. Secondary endpoints were further differences in allele and genotype frequency when additionally considering the allergy status in sense of atopy or metal allergy (i.e. patch test reactivity or LTT) in the two patient groups. We checked completed questionnaires for being properly filled in before transferring it to pseudonymized registration. Randomly chosen pseudonymized blood sample aliquots were processed for a repeated polymorphism analysis to assess consistency of results.

### Statistical analysis

Both, in the planning activity and the data evaluation the Institute for Statistics of the Munich University was officially involved. Data were recorded and further processed by use of the SPSS software (Version 23, IBM, Ehningen, Germany). Since this was the first, exploratory study i.e. there were no comparable studies respectively data on IL-1 polymorphisms and complicated arthroplasty we could not make a case number estimation. Furthermore, there were heterogeneous patient groups regarding type of arthroplasty and probably also implant alloy materials. However, we assumed that cytokine polymorphisms are person dependent, but do not depend on the type of implant. The statistical supervision and evaluation was done by two well qualified statisticians of the Institute for Statistics (Dr. T. Maierhofer and Prof. H. Kuechenhoff, head of the statistical consulting unit StaBLab) using cross tables with exact Fisher´s test with a allele model and also risk analysis (odds ratio) according to several authors (18–20).

## Results

### Questionnaire-aided history

Patients with complicated arthroplasty had reported various, sometimes multiple symptoms, most often pain (84.1%), reduction of range of motion (78.4%) and swelling (71.6%). With regard to the patient´s self-evaluation of their arthroplasty, the respective WOMAC Score (100 points mean perfect functionality) is also reflecting such symptomatic arthroplasty. In the arthroplasty control patients, the mean WOMAC Score was 92.13 ± 18.11. On the other hand, patients with complicated arthroplasty showed significantly lower mean WOMAC Score of 42.06±10.12. Furthermore, in 20 patients with complicated arthroplasty aseptic loosening was reported according to their physician´s diagnosis. There was a higher rate of atopy (i.e. presence of atopic diseases like allergic rhinocunjunctivitis, allergic asthma and/or atopic eczema) in the complicated arthroplasty group (24.5% vs 16.1%). There was also a higher number of patients with history of potential metal hypersensitivity in the complicated arthroplasty group (24.05% vs 9.7%). The results are summarized in **Table 3**.

**Table 3:** Results of the questionnaire, patch test and LTT in the patient groups (partly multiple positive reactions in the same patient).

		Arthroplasty patients with complaints (n=102)		Arthroplasty control patients (n=93)	
**Symptoms**	pain	96	94.1%	0	0%
	redness	15	14.7%	0	0%
	eczema	7	6.9%	0	0,%
	effusion	31	30.4%	0	0%
	swelling	73	71.6%	0	0%
	reduction of movement	80	78.4%	0	0%
	WOMAC Score (mean±Stdv)	42.06	±10.12	92.13	±18.11
	smoking (previously)	19	18.6%	16	17.2%
	smoking (current)	7	6.9%	4	4.3%
	smoking (overall)	26	25.5%	20	21.5%
**Atopy**	atopy (overall)	25	24.5%	15	16.1%
	hay fever	19	18.7%	11	11.8%
	asthma	13	12.7%	5	5.4%
	atopic eczema	0	0.0%	0	0.0%
	metal hypersensitivity (by history)	25	24.5%	9	9.7%
**Patch test**	nickel positive	20	19.6%	9	9.7%
	cobalt positive	7	6.9%	3	3.2%
	chromium positive	2	2.0%	1	1.1%
	gentamicin* positive	9	8.8%	0	0.0%
	benzoyl peroxide* positive	8	7.8%	2	2.2%
	HEMA* positive	2	2.0%	0	0.0%
	N,N-Dimethyl-p-toluidine* positive	2	2.0%	0	0.0%
**LTT**	nickel positive	29	28.4%	18	19.4%
	cobalt positive	3	2.9%	1	1.1%
	chromium positive	3	2.9%	0	0.0%

*bone cement component: 89 patients in the arthroplasty group with complaints and 31 control patients were patch tested to bone cement components because of a cemented arthroplasty; HEMA, hydroxy-ethyl-methacrylate.

### Patch testing

Positive metal patch test results in arthroplasty patients with complaints vs control patients were as follows: to Ni 19.6% vs 9.7%, to Co 6.9% vs 3.2%, to Cr 2.0% vs 1.1%. In total, 24 of the 102 symptomatic and 13 of the 92-symptom free arthroplasty patient was found to be metal allergic. Since only part of the individuals had cemented arthroplasty – in particular knee arthroplasty patients – a correspondingly lower number of patients had been tested to bone cement components. But still differences in overall reactivity were seen between the two groups (**Table 3**).

### LTT

When using the LTT to detect potential sensitization to Ni, Co or Cr, in general more sensitized individuals were found in the patients with symptomatic arthoplasty. 28.4% of the complicated arthroplasty patients had positive LTT to Ni, 2.9% to Co and 2.9% to Cr. In the control (i.e. asymptomatic) group the respective figures were: 19.4% to Ni, 1.1% to Co and none to Cr. The results of the questionnaire, patch test and LTT are also listed in **Table 3**.

### Genotyping

When assessing the frequency of the here selected three IL-1B polymorphisms and the IL-1RN intron 2 VNTR, the following results were obtained: There were no differences in the allele frequencies of the two different alleles C or T of the IL-1B polymorphism 3954. There was a slight trend for higher frequency of the C allele in the control group, especially in the patients without atopy, no positive patch test or LTT reaction. The results for IL-1B polymorphism 3954 are listed in **Table 4**.

**Table 4:** Allele frequencies of the IL-1B-3954 polymorphism including also subgroup analysis.

AlleleIL-1B3954		Symptomatic Arthroplasty patients (n=102)		Arthroplasty control patients (n=93)		p(Fisher´s exact test)
		Allele frequencies (n=204)	[%]	Allele frequencies (n=186)	[%]	
**Overall**	C	150	73.5	147	79.0	0.234
	T	54	26.5	39	21.0
**With atopy**	C	37	74.0	22	73.3	1.000
	T	13	26.0	8	26.7
**No** **atopy**	C	110	73.3	125	80.1	0.177
	T	40	26.7	31	19.9
**Patch test pos**	C	37	77.1	20	76.9	1.000
	T	11	22.9	6	23.1
**Patch test neg**	C	113	72.4	127	79.4	0.188
	T	43	27.6	33	20.6
**LTT pos**	C	41	70.7	26	72.2	1.000
	T	17	29.3	10	27.8
**LTT neg**	C	77	71.3	93	81.6	0.082
	T	31	28.7	21	18.4

Results of the Fisher´s exact test are given.

The IL-1B-31 polymorphism with the allele T had slightly higher frequencies in the control patient group (60.0% vs 52; n.s.). This overall tendency was more visible in the subgroup analysis of LTT positive individuals (70.6% vs 48.3%, p=0.05), but is unclear whether reflecting a more “protective” genotype. Results of the IL-1B-31allele frequencies are listed in **Table 5**.

**Table 5:** Allele frequencies of the IL-1B-31 polymorphism including also subgroup analysis.

Allele IL-1B 31		Symptomatic Arthroplasty patients (n=102)		Arthroplasty control patients (n=93)		p (Fisher´s exact test)
		Allele frequencies (n=204)	%	Allele frequencies (n=186)	[%]	
**Overall**	T	106	52.0	108	60.0	0.123
	C	98	48.0	72	40.0
**With atopy**	T	21	42.0	19	63.3	0.105
	C	29	58.0	11	36.7
**No** **atopy**	T	83	55.3	89	59.3	0.560
	C	67	44.7	61	40.7
**Patch test pos**	T	24	50.0	17	65.4	0.230
	C	24	50.0	9	34.6
**Patch test neg**	T	82	52.6	91	59.1	0.255
	C	74	47.4	63	40.9
**LTT pos**	T	28	48.3	24	70.6	0.050
	C	30	51.7	10	29.4
**LTT neg**	T	56	51.9	60	54.5	0.786
	C	52	48.1	50	45.5

Results of the Fisher´s exact test are given.

There were no relevant differences in the allele frequencies of the IL-1B-511 polymorphism. There was a trend for a higher frequency of the C allele of this polymorphism in the control group, but this was not statistically significant. The results for the IL-1B-511 polymorphism are listed in **Table 6**.

**Table 6:** Allele frequencies of the IL-1B-511 polymorphism including also subgroup analysis.

AlleleIL-1B511		Symptomatic arthroplasty patients (n=102)		Arthroplasty control patients (n=93)		p (Fisher´s exact test)
	Allele frequencies (n=204)	[%]	Allele frequencies (n=186)	[%]	
**Overall**	C	122	59.8	122	65.6	0.251
	T	82	40.2	64	34.4
**With atopy**	C	25	50.0	20	66.7	0.169
	T	25	50.0	10	33.3
**No** **atopy**	C	95	63.3	102	65.4	0.722
	T	55	36.7	54	34.6
**Patch test pos**	C	28	58.3	18	69.2	0.454
	T	20	41.7	8	30.8
**Patch test neg**	C	94	30.3	104	65.0	0.416
	T	62	39.7	56	35.0
**LTT pos**	C	33	56.9	27	75.0	0.083
	T	25	43.1	9	25.0
**LTT neg**	C	67	62.0	69	60.5	0.891
	T	41	38.0	45	39.5

Results of the Fisher´s exact test are given.

In the IL-1RN Intron 2 VNTR there was a much higher probability for the presence of arthroplasty related complaints correlated to the allele 498bp. In the control group of 93 symptom free individuals the allelle frequency of the 498bp allele was 10.8%, the SAE group had a frequency of 36.8%. This difference was highly significant (p<0.0001). With regard to the other allele frequencies of the IL-1RN Intron 2 VNTR polymorphism, we could not find differences. Interestingly this risk was further increased when the patients also had an atopic disease (p<0.000001) – but showed no significant link to patch test reaction or LTT reactivity. The results for the IL-1RN Intron 2 VNTR are listed in **Table 7**.

**Table 7:** Allele frequencies of the IL-1RN Intron 2 VNTR polymorphism including also subgroup analysis.

	Allele	Symptomatic arthroplasty patients (n=102)		Arthroplasty control patients(n=93)		p (Fisher´s exact test)
		Allele frequencies (n=204)	[%]	Allele frequencies (n=186)	[%]	
**Overall**	240bp	29	14.2	32	17.2	<0.0001
	326bp	7	3.4	9	4.8
	412bp	90	44.1	123	66.1
	**498bp**	**75**	**36.8**	**20**	**10.8**
	595bp	3	1.5	2	1.1
**With atopy**	240bp	4	8.0	5	16.7	<0.000001
	326bp	0	0.0	1	3.3
	412bp	15	30.0	21	70.0
	**498bp**	**30**	**60.0**	**3**	**10.0**
	595bp	1	2.0	0	0.0
**No atopy**	240bp	25	16.7	27	17.3	0.001
	326bp	7	4.7	8	5.1
	412bp	71	47.3	102	65.4
	**498bp**	**45**	**30.0**	**17**	**10.9**
	595bp	2	1.3	2	1.3
**Patch test pos**	240bp	2	4.2	2	7.7	0.008
	326bp	0	0.0	2	7.7
	412bp	24	50.0	20	76.9
	**498bp**	**21**	**43.8**	**2**	**7.7**
	595bp	1	2.1	0	0.0
**Patch test neg**	240bp	27	17.3	30	18.8	0.001
	326bp	7	4.5	7	4.4
	412bp	66	42.3	103	64.4
	**498bp**	**54**	**34.6**	**18**	**11.3**
	595bp	2	1.3	2	1.3
**LTT pos**	240bp	5	8.6	5	13.9	0.002
	326bp	3	5.2	1	2.8
	412bp	24	41.4	27	75.0
	**498bp**	**26**	**44.8**	**3**	**8.3**
	595bp	0	0.0	0	0.0
**LTT neg**	240bp	17	15.7	20	17.5	0.006
	326bp	4	3.7	8	7.0
	412bp	53	49.1	73	64.0
	**498bp**	**33**	**30.6**	**13**	**11.4**
	595bp	1	0.9	0	0.0

Results of the Fishers exact test are given. The Bonferroni correction was applied and resulted in a statistical significance p<0.01. The bold values are the statistical different values.

### Association analysis

**Figure 2:**
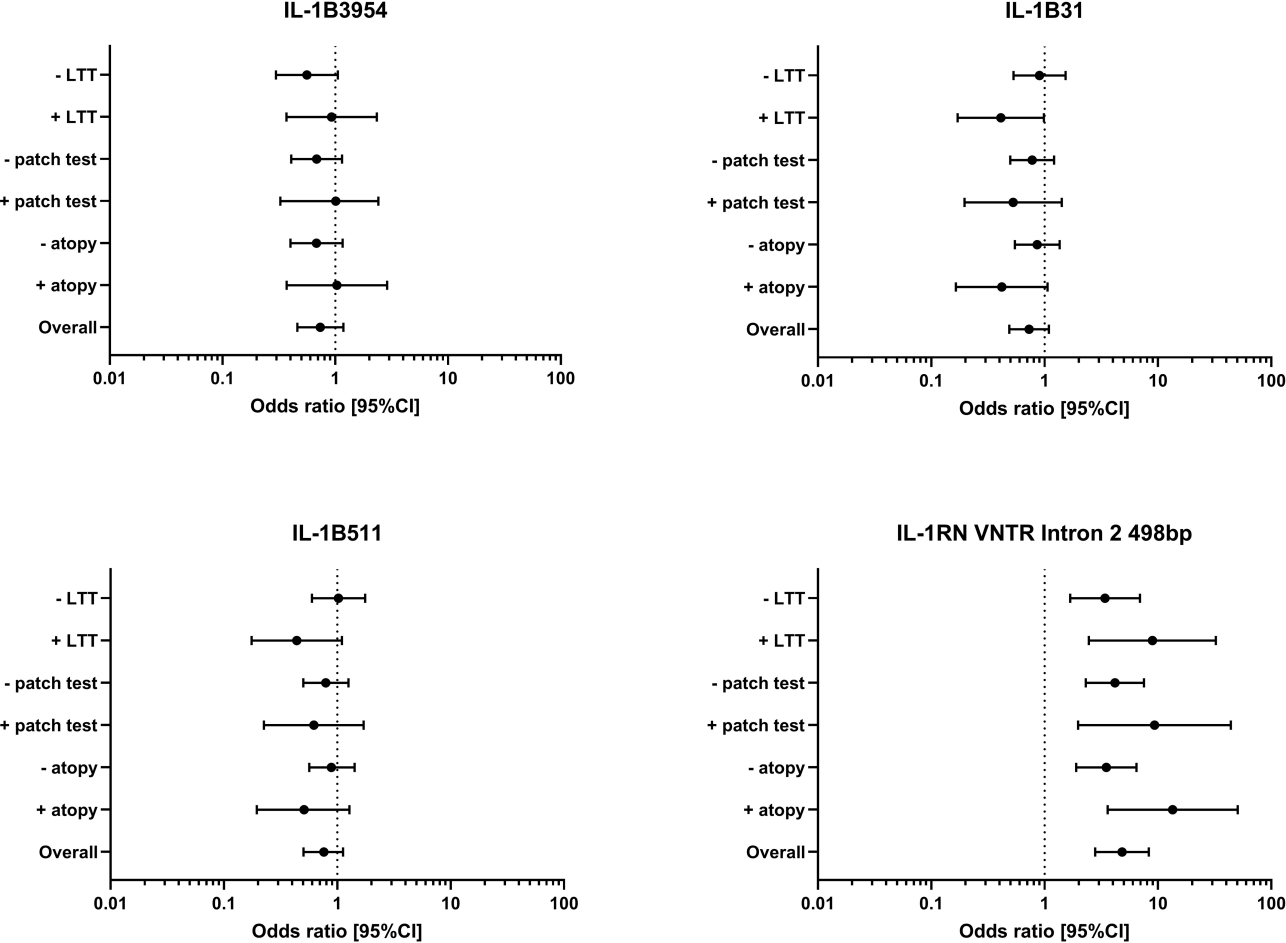
Odds ratio (and 95% confidence interval CI) of the IL-1B polymorphism IL-1B 3954, IL-1B 31, IL-1B 511 and the IL-1RN VNTR Intron 2 498bp allele are given for the risk analysis of the symptomatic arthroplasty patient group against the control group overall and also for the subgroups atopy (yes/no), patch test to metal (positive/negative) and LTT to metal (positive/negative).

We wondered if allele and genotype frequency were different when additionally considering the allergy status in sense of atopy or delayed type sensitization (i.e. patch test reactivity or LTT response). It must be mentioned that 54 of the 102 complicated arthroplasty patients did neither have patch test nor LTT reaction, i.e. no implant related delayed type sensitization was detected. For polymorphism IL-1B 3954 and IL-1B 511, subgroup analysis in patients with or without atopy, positive or negative patch test or LTT showed no relevant differences. IL-1B 31 polymorphism with the T allele was slightly more frequent in the arthroplasty control group. The subgroup analysis showed no differences for factors as atopy or patch test reactivity and for LTT positive status there was some influence by this polymorphism. Adjustment for such additional factors (e.g. atopy) was however significant for the IL-1RN Intron 2 VNTR when analyzing its relation to allele 498bp. Atopy alone had an association with the 498bp allele (non atopic: 30.0% versus atopic individuals 60.0% in the SA group). If concomitantly allele 498bp was present in atopic patients an over 4 times higher probability exists for the patients to have a symptomatic arthroplasty (**Figure 2**).

## Discussion

The identification of genetic traits regarding susceptibility for developing adverse reactions (,,intolerance “) to artificial joint replacement is confounded by a variety of factors. They include heterogeneity of clinical symptoms, difficulty in distinguishing other pathology like infection, potential genetic differences between populations and the potential limited influence of any single mutation on the disease features. Despite these limitations, we compared in this case control study caucasian patients with symptom free arthroplasty to patients with symptomatic joint replacement, in whom the referring orthopedic surgeons could not detect common problem elicitors like infection or mechanical causes (i.e. malalignement or malpositioning). In both patient groups, the allergy status and a selected series of polymorphisms of the IL-1 gene cluster were assessed. The degree to which the clinical heterogeneity of ,,adverse reactions”translates into different pathomechanisms including hypersensitivity is not yet known. However, several studies have pointed to a role of both (i) specific immune reactions in sense of hypersensitivity to metals and bone cement components and (ii) unspecific innate inflammatory mechanisms. For the latter the IL-1 genes are good candidates to mediate inflammatory response to ,,arthroplasty as foreign body “. A potential association between IL-1 gene cluster polymorphisms, for example IL-1B 3953, was reported in patients with dental implant failure (21) – but adequately powered prospective cohort studies are missing. On the other hand, IL-1B polymorphisms, namely IL-1B 3953 and IL-1B 511and mutations in IL-1RN (like IL-1RN*2) were found to influence systemic and local inflammation in the autoinflammatory diseases (22, 23). For several polymorphisms in the IL1B locus (23, 24), in the IL1RN VNTR (25) and also RANK, KREMEN2 among others associations with osteoarthritis were described (9). These functional and linkage data prompted our analysis of the IL-1 gene cluster, of which we genotyped three polymorphisms of the IL-1B gene (IL-1B-3954, IL-1B-31 and IL-1B-511) and one of the IL-1-RN (Intron 2 VNTR).

We observed an association between carriage of the 498bp allele of the IL-1RN (Intron 2 VNTR) and development of clinical symptoms (controls versus symptomatic patients: 10.8% versus 36.8%). Furthermore, when stratifying the patients for the presence of atopy, the risk for a complicated arthroplasty in patients with the 498bp allele of the IL-1-RN (Intron 2 VNTR) was over 4 times higher.

The particle induced response in patients with metal arthroplasty depends from individual genetic susceptibility. Several genes play a role as for example KREMEN2, RANK, OPG and others (9). Patients with polymorphisms in these genes had a higher risk of developing osteolysis around the metal implant. Polymorphisms in inflammatory cytokines as TNFa and IL-6 or growth factors as TGFbeta have been reported to be associated with aseptic loosening (26, 27). In their review Del Buono et al. (28) concluded, that multiple genetic polymorphisms are likely to be associated with an increased risk of aseptic loosening. Consistent with that concept, the contribution of single polymorphisms might be small and a series of other factors like Toll-like receptor activation or bacterial pathogen associated molecular patterns might influence the interaction ,,organism – implant “ (29). Surely, there is often a gene network which is responsible for an effect as it was shown for Crohn´s disease (30, 31). On the other hand, it seems that IL-1B polymorphisms may not have a clearcut association with rheumatoid arthritis (32). With regard to osteoarthritis (OA) an association with predisposition for hip radiographic osteoarthritis was described for the heterozygous and homoygous carriers of the IL-1B allele -511T (24). Increased IL-1B gene expression in peripheral blood leukocytes was reported in a subset of knee OA patients with increased pain and higher risk of radiographic progression of OA (33). In the symptomatic patients in our study – despite the heterogeneity of symptoms – at least periimplant infection was excluded by the referring physicians, and all were caucasians. In our here presented patients, several aspects were questioned: 1) A ,,general” role of allergy background (here atopy, i.e. presence of allergic rhinoconjunctivitis, allergic asthma, atopic eczema) – as suggested by the observation of Graves et al. (34); 2) the specific allergic delayed type sensitization to implant components like metals (by patch test and LTT) – considering however that there is no general linear cause-effect relationship; 3) potential genetic traits regarding susceptibility for developing symptomatic arthroplasty.

Increased rates of patch test reactivity to metals and bone cement components were found in the symptomatic patients. However, the majority in the symptomatic patients was without such sensitization and some symptom free patients (to a lesser extent) did also have such positive cutaneous patch test reactions. This observation fits to the assumption, that metal implant allergy is a rare event and remains a diagnosis of exclusion by differential diagnostics (35), since patients with cutaneous metal allergy might even well tolerate the respective metal alloys when implanted. In addition, the role of delayed type hypersensitivity (DTH) to metals may be complicated by different interpretation of the term “DTH” by allergists and surgeons. Langton et al. reported in 2022 that variation in HLA class II phenotype influences susceptibility to DTH following implantation of MoM CoCr hip prosthesis (36). Patients who underwent revision of their MoM hip prosthesis had: assessment of metal ion concentration in blood, serum, joint fluid; assessment of explanted joint for volumetric wear; evaluation of periimplant tissue samples; HLA genotyping from buccal samples. Prove of DTH was defined by a particular periimplant histology showing perivascular lymphocytic infiltrates (“ALVAL”). It however remains problematic that such parameter rather describes “lymphocyte-dominated” infiltrates (and no vasculitis) than its functional meaning. The drawback of such terminology was discussed few months earlier in an international histopathology review by Perino G et al. (37) and the authors propose to omit the term “ALVAL” and rather use the terms ALTR/ARMD. When further evaluating presence of “atopy” and detection of delayed type sensitization (either by patch testing or LTT) in our patients, both aspects were not connected. This is in accordance with many studies – summarized by R Spiewak – pointing as well to the non-existing relationship between atopy and contact hypersensitivity (38) This might help to explain, why presence of delayed type sensitization did not have significant influence – but in the presence of atopy, the potential for a complicated arthroplasty in patients with the 498bp allele of the IL-1-RN (Intron 2 VNTR) was over 4 times higher. In human inflammatory skin diseases IL-1RN gene polymorphisms are discussed in association with allergic contact dermatitis and psoriasis (39). When looking on atopic eczema, we found no such investigations. With regard to allergic rhinitis, the study of Veli-Pekka et al. focused on the role of IL-1 polymorphisms comparing healthy and allergic rhinitis individuals. It was stated that differences in genotype distribution “was mainly due to an increased number of IL1A allele G homozygotes and IL1RN allele 2 homozygotes in allergic rhinitis” (40). In allergic asthma, the third atopic disease, the experimental benefit from administration of recombinant IL-1Ra has been described. However, we did not find reports with regard to atopic diseases on our here described finding, i.e. *IL-1RN* Intron 2 polymorphism at allele 3.

In addition, we did not find an investigation that did describe the significance or changes of activities of this variant – inter alia whether this polymorphism results for example in IL-1Ra protein that is potentially less secreted, altered or with less affinity to the IL-1 receptor.

Our present study does have some limitations. First, the two patient groups both have CoCrMo-based arthroplasty, but in the group of complicated arthroplasty there are predominantly TKR-patients, i.e., patients being per se more dissatisfied with their arthroplasty (3). Nevertheless, genetic background – here in sense of IL-1 polymorphisms – is independent from the type of arthroplasty and should still be assessable by comparing the two groups. Secondly, our study might be limited by the involvement of many treating orthopaedic surgeons and their presumably slightly different implantation methods. However, their diagnostic protocols to detect “problem elicitors” before referring the patients to the allergy diagnostics were comparable. Third, we did not analyse further patient characteristics, such as potentially confounding comorbidities, pre-arthroplasty condition of the respective joint or socioeconomic status as potential factors contributing to dissatisfaction following TKR. Fourth, we have no follow up data like results of revision surgery including serological or histopathological data, clinical course and use of “hypoallergenic material” upon revision.

In conclusion, our study provides encouraging data for a role of cytokine (here IL-1) -polymorphisms as contributing factor in complicated total joint replacement. Up to our knowledge, this is the first report on patients with symptomatic arthroplasty, in which IL-1B polymorphisms were assessed and significant associations were found in particular with one allele of the IL-1-RN (Intron 2 VNTR).

This association (,,risk profile “) was independent from contact (delayed type) allergy, but was in particular with regard to IL1-RN (Intron 2 VNTR) additionally increased by the simultaneous presence of atopic disease. Larger cohort studies should be performed to corroborate the here described findings regarding their clinical relevance.

## Data availability statement

The original contributions presented in the study are included in the article/supplementary materials, further inquiries can be directed to the corresponding author/s.

## Ethics statement

The studies involving human participants were reviewed and approved by Ethical commission of the LMU Munich, Pettenkoferstr. 8a D-80336 Munich, GErmany. The patients/participants provided their written informed consent to participate in this study.

## Author contributions

BS conceived and designed the study. PT, DL, KR, AS, IB, acquired the clinical data. BS and DL performed the laboratory experiments. BS analysed the data and interpreted the data in collaboration with TM and all other authors. BS and PT wrote the first draft of the manuscript. SE revised critically the first draft and gave helpful comments. All authors contributed to the article and approved the submitted version.

## Funding

CeramTec, Plochingen, Germany. The funder was not involved in the study design, collection, analysis, interpretation of data, the writing of this article or the decision to submit it for publication.

## Acknowledgments

The authors would like to thank Ralph Pohl for his valuable technical assistance.

## Conflict of interest

The authors declare that the research was conducted in the absence of any commercial or financial relationships that could be construed as a potential conflict of interest.

## Publisher’s note

All claims expressed in this article are solely those of the authors and do not necessarily represent those of their affiliated organizations, or those of the publisher, the editors and the reviewers. Any product that may be evaluated in this article, or claim that may be made by its manufacturer, is not guaranteed or endorsed by the publisher.
